# Enrichment of a mixed microbial culture for PHA production with used cooking oil as substrate

**DOI:** 10.1007/s00449-026-03359-x

**Published:** 2026-07-09

**Authors:** C. Ucha, D. Correa-Galeote, A. Val del Rio, A. Pedrouso, A. Mosquera-Corral

**Affiliations:** 1 Department of Chemical Engineering, School of Engineering, CRETUS, Universidade de Santiago de Compostela, Santiago de Compostela, Spain; 2 Microbiology Department, Faculty of Pharmacy, Institute of Water Research, University of Granada, Granada, Spain

**Keywords:** COD/N ratio, Fatty acids, Pulsed feeding, Waste valorisation, Withdrawal strategy

## Abstract

The synthesis of polyhydroxyalkanoates (PHA) by a mixed microbial culture (MMC) employing used cooking oil (UCO) as substrate represents a promising bioprocess for organic wastes valorisation. In this study, MMC enrichment in a sequencing batch reactor (SBR) was investigated using UCO without pretreatment as the sole carbon source. Two SBRs (SBR1 and SBR2) were operated under a feast-famine regime with uncoupled carbon and nitrogen supply, differing in the timing of the biomass withdrawal and nutrient filling. In SBR1, with effluent withdrawal at the end of the feast phase, better poly(3-hydroxybutyrate-*co*-3-hydroxyvalerate) (PHBV) accumulation was achieved (average values of 4.2 ± 1.6 wt. %, maximum of 19.9 wt. %) compared to SBR2, with withdrawal at the end of the famine phase (average values of 2.7 ± 1.1 wt. %, maximum of 5.2 wt. %). The highest PHBV accumulation in SBR1 was obtained at a COD/N ratio of 46 g/g, with an active biomass concentration of approximately 1 g VSS/L. Additionally, a single UCO pulse enhanced substrate hydrolysis and consumption compared to multiple pulses. Microbial community analysis revealed the enrichment of bacterial OTUs with PHA accumulation capacity related to *Azospirillum*, *Acinetobacter*, *Gordonia*, *Burkholderia*, and *Bacillus*. Among fungal OTUs, *Geotrichum*, *Meyerozyma*, and *Pascua* were the most abundant. Multivariate statistical analysis indicated that the synergistic interaction of *Burkholderia* and *Chitinophaga* played a crucial role in achieving the highest PHBV accumulation levels.

**Graphical abstract:**

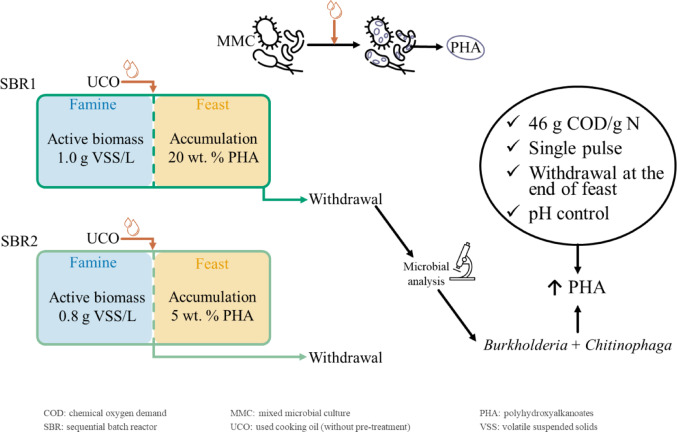

**Supplementary Information:**

The online version contains supplementary material available at 10.1007/s00449-026-03359-x.

## Introduction

Bio-based materials production plays a crucial role in reducing environmental impact and supporting the concept of the circular economy, which has gained interest in the last decades. Among these materials, polyhydroxyalkanoates (PHA) have the potential to replace widely used petrochemical plastics. PHAs are polymers biosynthesised by different microorganisms in response to an excess of carbon combined with the limitation of a nutrient in the reaction medium. In the biological process, this polymer serves as a reservoir of carbon and energy that the microorganisms use to growth once the limited nutrient (usually nitrogen or phosphorous) becomes available again in the medium [1–3]. PHAs are non-toxic, biodegradable materials (in soil, water, and compost) [4–6], and biocompatible. Among PHA monomers, 3-hydroxybutyrate (3HB) is commonly produced by microorganisms, whereas 3-hydroxyvalerate (3HV), although less frequently produced, can be incorporated into PHA. The copolymer poly(3-hydroxybutyrate*-co-*3-hydroxyvalerate) (PHBV) exhibits physicochemical properties that are strongly influenced by the relative proportion of these two monomers. This copolymer has a lower degree of crystallinity and enhanced flexibility and mechanical strength compared to 3HB monomer [7], these characteristics make PHBV a particularly attractive biopolymer for certain high-value applications [8].

The biological synthesis of PHA could be carried out using either a pure or a mixed microbial culture (MMC). Pure cultures have traditionally been used for these studies due to the ease of selecting operating conditions and their better performance, achieving high concentrations of cell dry weight (4–50 g/L) [9–11]. However, they require sterile conditions to avoid contamination and are more sensitive to environmental changes [12]. On the other hand, MMCs, which consist of diverse microbial communities, offer enhanced adaptability and resilience to environmental changes. For this reason, PHA production by MMC has gained attention in recent years [13–16].

In addition to the use of MMCs, a sustainable and cost-effective approach for PHA production also involves the use of waste substrates. Among the possible waste streams, used cooking oil (UCO) stands out, as it can be directly used without prior acidification, unlike other wastes that need to be converted into volatile fatty acids (VFAs) before feeding. UCO is mainly composed of triacylglycerides, along with smaller amounts of diacylglycerides and free fatty acids. Therefore, when PHA is synthetised from UCO, the main metabolic pathway involved is β-Oxidation. Prior to uptake, extracellular hydrolysis is required to release glycerol and fatty acids, which are then transported into the cells. Once inside the cell, fatty acids are metabolized via β-Oxidation, generating intermediate compounds that can be used for PHA synthesis by various enzymes.

From a resource availability perspective, UCO represents an abundant and underutilized waste stream. UCO is a lipidic waste globally produced, approximately 200 Mt are produced annually worldwide, with 15 Mt coming from the United States, 24 Mt from Asia, and 4 Mt from the European Union [17]. In Europe, the selective collection of UCO is mandatory, and management companies play a key role in ensuring its origin and quality under strict regulatory frameworks governing its treatment. However, household UCO remains difficult to manage, and its collection should be actively promoted by local authorities. Furthermore, its variable quality necessitates proper classification [18]. In Spain, an average of 300 kt of UCO are produced annually; however, only 5 % of the household UCO is recovered for further valorisation [19]. Nevertheless, in Spain UCO is subject to separate collection from other waste streams, and oils with different characteristics must not be mixed with each other or with other substances [20].

The valorisation of UCO for PHA synthesis with pure cultures or genetically modified bacteria has been widely studied, with reported productivities ranging from 0.29 to 1.73 g PHA/(L·h) [21, 22]. Specifically, PHA production from UCO has been demonstrated using pure cultures cultivated mainly in flasks at small scale, achieving concentrations between 0.67 and 1.2 g PHA/L [9, 10, 21]. However, limitations in hydrolysis, transport, or metabolic conversion of UCO may result in incomplete utilization of its fatty acids, leading to their accumulation either in the medium or within the cells. These limitations become even more critical when using MMCs.

To overcome these limitations, several studies have proposed the conversion of UCO, via hydrolysis and/or acidogenic fermentation, into short-chain organic acids that are subsequently fed to a MMC enriched under a feast-famine regime [23, 24]. Furthermore, a pretreatment step can be included, such as saponification, to favour UCO hydrolysis in the liquid media [25, 26] and to obtain adequate MMC enrichment and PHA accumulation. The main limitations of these studies, particularly when considering implementation and scale-up, include the risk of contamination in pure cultures and the high cost associated with potential UCO pretreatment required for efficient bacterial utilization.

By contrast, the direct use of raw UCO for the enrichment of MMCs, without prior hydrolysis, emulsification, or conversion to free fatty acids, has scarcely been reported. One of the few targeted studies reported that direct MMC enrichment on PHA-storing bacteria using raw UCO was hindered by filamentous overgrowth, and that it was necessary to perform a previous selection of the MMC using a synthetic substrate different from the raw UCO [27]. Overall, the direct MMC enrichment with raw UCO is challenging, even though microorganisms are indeed capable of directly utilizing the fatty acids contained in UCO for PHA production [28].

The objective of this study is to evaluate the enrichment of a MMC derived from activated sludge for PHA synthesis using raw UCO as the carbon source, without any pretreatment. In addition, the effects of the COD/N ratio and the biomass withdrawal strategy were evaluated. The dominant microbial populations (bacteria and fungi) in the MMC were also analysed to understand how changes in reactor operational conditions influence them.

## Materials and methods

### Experimental conditions for the enrichment reactors

To enrich the MMC with accumulating microorganisms, two lab-scale sequencing batch reactors (SBR1, 10 L, and SBR2, 2 L) were operated. Two selection strategies were applied in both reactors to promote the enrichment, the aerobic dynamic feeding (ADF) [29] and the double growth limitation (DGL) [30]. The ADF was designed to impose a feast-famine regime with 12-hour cycles, comprising 6 h of feast phase followed by 6 h of famine phase. The DGL consisted of supplying the carbon source (raw UCO) either in single or multiple pulses to impose a feast phase. The nitrogen source (NH_4_Cl) was added 6 h later, assuming that UCO had already been consumed, marking the start of the famine phase.

The difference between the two SBRs was the effluent withdrawal and filling with nutrient solution, in SBR1 occurred at the end of the feast phase, whereas in SBR2 at the end of the famine phase. The volume exchange ratio was 50 % for both, resulting in hydraulic and solids retention times of 1 day. Details of the operational cycle distribution for each SBR is available in Table S.1.

Aeration was continuously supplied to both reactors using an air pump (Laboport N 86 KTP, KNF Neuberger, USA), connected to diffusers placed at the bottom of the reactor, providing dissolved oxygen for the biological reactions and mixing of the biomass inside the reactor, removing the need for a mechanical stirrer. The temperature was maintained at 30 °C using a thermostatic bath (Tectron Bio-100, JP Selecta, Spain) connected to a thermal jacket. The pH inside the SBRs was not actively controlled; however, the nutrient solution was adjusted to 7.0 ± 0.5 using KH_2_PO_4_ and NaHCO_3_. From day 240 onwards, SBR1 was equipped with a pH controller (42 Series, Chemitec, Italy) connected to two peristaltic pumps, which supplied HCl (0.5 M) or NaOH (0.5 M) to maintain the reactor medium at pH values around 7.0 ± 0.3.

### Inoculation and operational stages

Activated sludge from the municipal wastewater treatment plant of Santiago de Compostela (Northwest Spain) was used as the inoculum for both SBRs, with a concentration of solids in the reactors of 2.5 ± 0.3 g VSS/L (ratio VSS/TSS of 0.73 ± 0.01 g/g). Activated sludge typically exhibits high microbial diversity and abundance, providing a suitable inoculum for the selective enrichment in PHA-storing microorganisms.

SBR1 (withdrawal at the end of the feast phase) was operated for 500 days, divided into three stages (Table 1). The first stage (S-I, days 0–203) was operated with the addition of three UCO pulses, an increment of the organic loading rate (OLR) and a fixed nitrogen loading rate (NLR). In the second stage (S-II, days 204–253), the OLR and NLR were maintained, but the UCO was added in a single pulse instead of three. Finally, in S-III (days 254–500), the UCO was added in a single pulse, the OLR suffer small variations, while the NLR was decreased and slightly modified to adjust its value and avoid the excess of nitrogen at the end of the famine phase.

**Table 1:** Summary of operational parameters and feeding strategies applied in SBR1 and SBR2 throughout the enrichment process

Stage	SBR1			SBR2
	S-I	S-II^b^	S-III	
Operational days	0–203	204–253	254–500	0–188
UCO pulses/cycle	3	1	1	1
UCO added (mL/pulse)	0.9–1.3	4.0	4.0–4.2	0.7
OLR (g tCOD/(L·d))	1.2–1.8	1.8	1.8–1.9	1.5
tCOD (mg/L) ^a^	590–900	900	900–934	736
NLR (mg N/(L·d))	78.5	78.5	36–46	32.6
NH_4_^+^-N (mg N/L) ^a^	40	40	18–23	16
COD/N (g/g)	15–23	23	45–52	46

*COD:* chemical oxygen demand, *COD/N:* COD fed at the feast phase divided by the nitrogen fed at the famine phase, *NLR:* nitrogen loading rate, *OLR:* organic loading rate, *UCO:* used cooking oil

^a^Concentrations of UCO (as tCOD, feast phase) or nitrogen (famine phase) in the reactor just after feeding in each cycle

^b^A control of pH was implemented in SBR1 at day 240

SBR2 (withdrawal at the end of the famine phase) was operated for 188 days under conditions like S-III of SBR1 (Table 1): fed in a single pulse with fixed OLR and NLR.

### Feeding composition

Both SBRs were fed with: (i) raw UCO (carbon source) at the beginning of the feast phase; (ii) NH_4_Cl solution (nitrogen source) at the beginning of the famine phase; (iii) nutrient solution at the beginning of the famine phase in SBR1 and at the beginning of the feast phase in SBR2 (Table S.1). This feed distribution defines the feast phase (UCO addition for PHBV accumulation) and the famine phase (nitrogen addition for biomass growth).

The raw UCO was collected from the university’s hostelry service and used as the organic substrate without pretreatment. It was characterized by its content in fatty acids (93.84 ± 2.34 %), organic matter (2.63 ± 0.29 g tCOD/g UCO), elemental composition (CH_1.93_O_0.11_N_0.002_), and biodegradability (58.47 ± 2.2 %), see more details in Table S.2.

The nutrient solution (pH 7.0 ± 0.5) comprised KH_2_PO_4_ (0.3 g/L), MgSO_4_ (0.0099 g/L), KCl (0.066 g/L), NaHCO_3_ (0.30–0.45 g/L), allylthiourea (0.0044 g/L) and a micronutrient solution (1 mL/L) described by Vishniac and Santer [31]. Additionally, in SBR1, the nutrient solution contained NH_4_Cl (0.3 g/L in S-I and S-II and 0.17 g/L in S-III). In SBR2, NH_4_Cl was added separately as 100 mL of a 1.25 g NH_4_Cl/L solution. All chemicals and reagents used throughout the reactor operation are specified in Table S.3.

### Sampling and analytical methods

In both SBRs, two samples per cycle were analysed (three times per week), collected at the end of the feast and famine phases. All the samples were analysed in duplicate. To characterise the liquid fraction, the samples were centrifuged (7500 rpm for 10 min) and then filtered using a 0.45 μm pore size mixed cellulose ester membrane (Advantec, Japan). The soluble chemical oxygen demand (sCOD) [32] and total nitrogen (TN) concentrations (TOC-L analyser with the TNM-module, TOC-5000 Shimadzu, Japan) were determined in the liquid phase. Concentrations of total COD (tCOD) [32], total suspended solids (TSS) and volatile suspended solids (VSS) [32], as well as PHBV and fatty acids contents were measured in the solid phase. The pH was measured with a pH & Ion-Meter model GLP 22 (Crison, Spain). The dissolved oxygen concentration was continuously monitored inside the reactors every 5 min using a portable multimeter (HQ40d, Hach-Lange, USA).

PHBV and fatty acids quantification were performed using the method described by Smolders et al. [33], with slight modifications. Two biomass samples were collected at each measurement point and dried at 50 °C in an oven (Memmert BE300, Memmert, Germany) for 24 h. Then, 15–20 mg of the dried sample were weighed into glass tubes. A mixture of HCl:1-propanol for acid digestion, 1,2-dichloroethane for extraction, and 1-propanol benzoic acid as the internal standard were added. Subsequently, the samples were digested at 100 °C for 3 h (Conterm, JP Selecta, Spain). After digestion, 3 mL of distilled water were added to facilitate liquid-liquid extraction. Following vortex mixing and phase separation, the organic phase was transferred to a vial for bioproduct quantification by gas chromatography (GC).

A GC system (Agilent Technologies 6850 Series II, Agilent, USA) equipped with a flame ionization detector (FID) was used for analysis. Each sample was analysed using duplicate injections. PHBV and fatty acids were separated on an HP-INNOVAX capillary column (Agilent, USA), with helium as the carrier gas at a flow rate of 1 mL/min. The split injection and detector temperatures were set at 250 °C and 275 °C, respectively. The initial temperature was 50 °C for 2 min, followed by a 10 °C/min ramp to 250 °C, maintained for 8 min. Data analysis was performed using the OpenLab CDS Chemical station software (Agilent, USA). Calibration curves were obtained using commercial standards of PHBV copolymer (3HB, 90.82 % w/w, and 3HV, 9.18 % w/w), fatty acids (Palmitic, Stearic, Oleic, and Linoleic), and benzoic acid as an internal standard.

### Calculations

The OLR (as g tCOD/(L·d)) and the NLR (as mg N/(L·d)) were calculated as the amount of UCO added, in terms of tCOD, or the amount of nitrogen added, as mg N, to the reactor per cycle, considering the number of cycles per day (2) and the volume of the reactor (Vr).

$$\:OLR=\frac{UCO\:\left(g\:tCOD/cycle\right)\times\:2\:(cycles/day)}{Vr\:\left(L\right)}$$

$$\:NLR=\frac{Nitrogen\:\left(mg\:N/cycle\right)\times\:2\:(cycles/day)}{Vr\:\left(L\right)}$$

The COD/N ratio was determined as the tCOD fed at the feast phase divided by the nitrogen fed at the famine phase. The active biomass (X) was determined at the end of the famine phase as the solid concentration (g VSS/L) minus the remaining concentration of bioproducts in the microbial cells (PHBV and fatty acids in g/L) [34]. The PHBV and fatty acids contents were expressed in terms of dry weight % (wt. %), referred to the mass of total suspended solids.

$$\:Content\:\left(wt.\:\%\right)=\frac{Measured\:mass\:ofPHA\:or\:Fatty\:acids\:\left(g\right)}{TSS\:in\:the\:medium\:\left(g\right)}\times\:100$$

### Microbiological analysis

DNA was extracted from three independent replicated biomass samples taken from SBR1 using the FastDNA-2 mL SPIN Kit for Soil method, which was briefly modified, and the FastPrep24 instrument (MP-BIO, USA). The diversity of bacterial and fungal communities was characterized using Illumina sequencing with the primers Pro341F/Pro805R [35] for bacteria and FungiQuantF/FungiQuantR [36] for fungi. The operational taxonomic units (OTUs) were defined at a 97 % similarity threshold from the raw sequence data using the MothurMiSeq pipeline and Mothur software version 1.44.1 [37]. Taxonomic classification of *Bacteria* and *Fungi* was conducted utilizing the custom BLAST tool in Geneious version 2025.1.2 (Biomatters, New Zealand), comparing sequences against the NCBI bacterial 16 S rRNA and fungal 18 S rRNA databases [38].

A non-metric multidimensional scaling (NMS) analysis was used to link the structure of the bacterial and fungal communities to the operational parameters by means of the PC-ORD software (Wild Blueberry Media, Corvallis, OR, USA).

## Results and discussion

### Dynamics of biomass concentration during the enrichment of the MMC

**Fig. 1:**
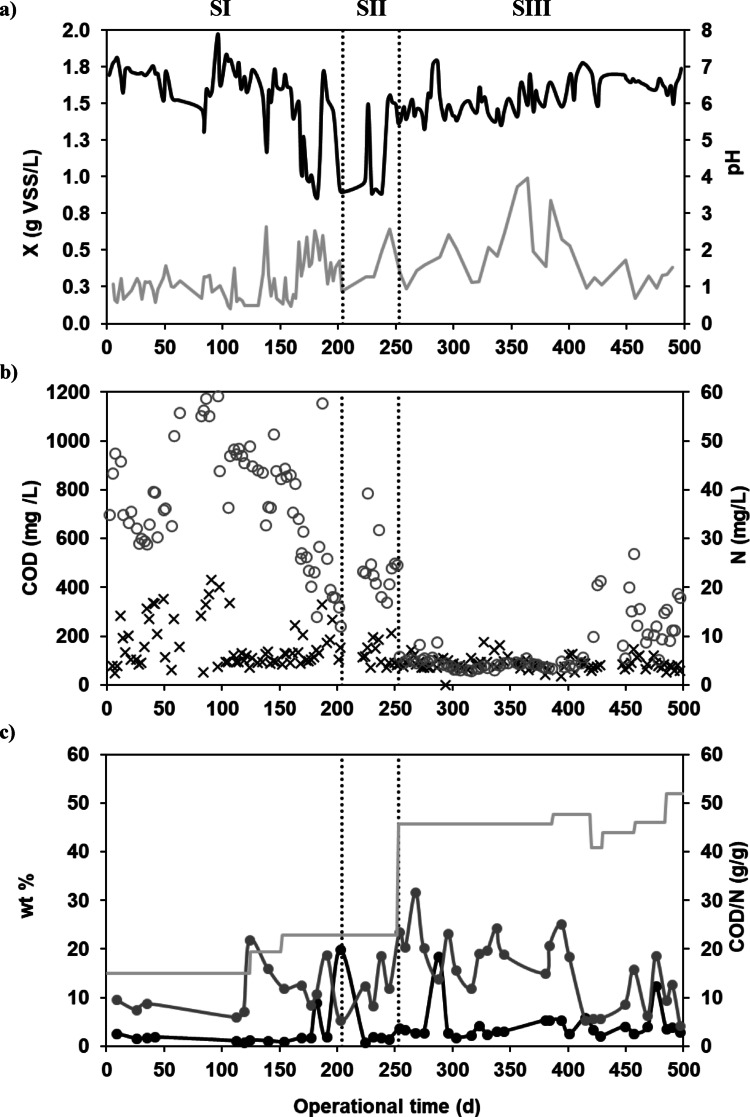
Evolution throughout the operational time of different parameters in SBR1: **a** concentration of active biomass X (), and pH value () at the end of the famine phase; **b** concentrations of sCOD at the end of the feast phase (×) and of N at the end of the famine phase (○); **c** percentages of accumulated PHA () and fatty acids () at the end of the feast phase and COD/N ratio () fed through the operation. The different stages are defined with a pointed vertical line ().

In SBR1, the fed COD and N concentrations were adjusted to identify a suitable COD/N ratio for MMC enrichment and active biomass growth. The VSS/TSS ratio consistently remained around 0.94 ± 0.03, which indicates that most of the solids were organic, although a minor inert fraction may still have been present. The active biomass concentration started low during the first 125 days (S-I) with values ranging from 0.1 to 0.4 g VSS/L, while the PHBV and fatty acids contents were 1.55 ± 0.52 and 10.41 ± 4.05 wt. %, respectively (Fig. 1). The increase in COD fed, from 590 to 900 mg tCOD/L, led to a rise in active biomass concentration, reaching 0.4–0.6 g VSS/L between days 150 and 203 (S-I). Following this change, PHBV content attained peaks of 9 wt. % (day 182) and 20 wt. % (day 203), whereas fatty acids content increased up to 20 wt. %. The observed rise in PHBV content after day 150 was likely associated with the lower nitrogen concentration during the feast phase, as it had been previously consumed for biomass growth during the famine phase, resulting in effective nutrient limitation [39].

However, the nitrogen added in stages S-I and S-II (40 mg N/L inside the reactor) was not completely consumed during the famine phase and remained still available in the subsequent feast phase (Fig. 1b), hindering PHBV accumulation instead of promoting it [40]. Therefore, the nitrogen concentration fed during the famine phase was halved in stage S-III (Table 1), leading to an increase in active biomass and bioproducts accumulation, with peak values of 18.5 wt. % PHBV (day 288) and 31 wt. % fatty acids (day 268).

The highest concentrations of active biomass (up to 1 g VSS/L) were achieved between days 330 and 400 (S-III), where the nitrogen fed (20 mg/L inside the reactor) was largely consumed during the famine phase (Fig. 1b). Although these values are lower than those reported by other authors using UCO [22, 41], where total biomass concentrations of 54 and 115 g/L were achieved, those studies employed a hydrolysed UCO and pure strains in a high-cell density reactors, thereby eliminating the need for culture enrichment and mitigating limitations associated with the carbon source.

From day 420 onwards, several minor adjustments to the nitrogen concentration were tested (Fig. 1c) to enhance PHBV accumulation; however, these were unsuccessful and nitrogen was not fully consumed during the famine phase. Therefore, towards the end of the operational period (days 400–500, S-III), the active biomass decreased to approximately 0.32 ± 0.09 g VSS/L (Fig. 1a). This result highlights the sensitivity of the enrichment process with raw UCO, as well as the importance of properly controlling nitrogen feeding.

In SBR1, the increase in active biomass concentration was achieved first by increasing the COD concentration fed during the feast phase, and then by decreasing the nitrogen concentration to restrict its availability during the feast phase. Among the COD/N ratios assayed, the most suitable for promoting an increase in active biomass (up to 1 g VSS/L) was approximately 46 g tCOD/g N (Fig. 1c), which is equivalent, according to the UCO elemental composition (Table S.2), to approximately 13 g C/g N. This value falls within the range of other reported values obtained from similar enrichment processes (ADF) using no pre-fermented substrates such as crude glycerol (15.9 g C/g N) [42] or UCO as the substrate (10–20 g C/g N) [21, 27], but with pure cultures. In addition, Zeng et al. [43], using a mixture of VFAs as a carbon source, observed that C/N ratios in the range of 40–80 g C/g N favored PHA accumulation at the expense of biomass growth, while lower ratios (5–20 g C/g N) enhanced biomass proliferation but restricted PHA synthesis. Therefore, the selection of the feed C/N ratio will help to enrich the MMC.

In addition, to maintaining an appropriate COD/N ratio, efficient hydrolysis of raw UCO is essential. Inadequate hydrolysis restricts the availability of soluble substrates for microbial uptake, thereby limiting both biomass growth and PHBV accumulation. For this reason, the soluble COD in the liquid phase was monitored at the end of the feast phase (Fig. 1b). During the first 100 days, values remained relatively high (200–400 mg sCOD/L). As the active biomass concentration increased, they decreased to approximately 100 mg sCOD/L and subsequently stabilized for the remainder of the operation. These results indicate that the MMC have required approximately 100 days to establish a stable UCO hydrolysis, enabling efficient substrate consumption.

### Feeding strategy: multiple or single pulses

Pulsed feeding strategies have been reported to reduce substrate inhibition by the carbon source [44, 45] and to promote high PHA storage [34]. Considering the low solubility of UCO and the potential inhibition caused by free fatty acids, a pulsed feeding strategy (three pulses during the feast phase) was implemented in stage S-I of SBR1 to facilitate the hydrolysis of raw UCO. During this stage the dissolved oxygen (DO) concentration profile showed a marked decrease after each UCO feeding pulse (first pulse from 7 to 4 mg O_2_/L; second pulse from 6 to 2 mg O_2_/L; third pulse from 5 to 1 mg O_2_/L at day 126) followed by a subsequent recovery once the substrate was consumed (Fig. S.1a). Indicative of an efficient substrate uptake and suggesting a good metabolic activity of the MMC, despite the potential inhibitory effects of free fatty acids contained in the raw UCO.

To enhance the UCO consumption and favour more stable PHBV accumulation, in S-II a single pulse feeding strategy was implemented from day 204 onwards (Table 1), maintaining the same total raw UCO fed (900 mg tCOD/L). With a single pulse, a longer time for UCO hydrolysis and assimilation during the feast phase is allowed. As shown in Fig. S.1b (day 225), the DO concentration decreased from almost 5 to 2 mg O_2_/L during the first hour after the addition of raw UCO, rising again to 5 mg O_2_/L before the effluent withdrawal phase, confirming the capacity of the MMC to metabolize the raw UCO, even using a single pulse.

The average PHBV content achieved with a single pulse increased progressively from 2 wt. % (day 231) to 12 wt. % (day 476), with a peak of 18.5 wt. % (day 288). The fatty acids content in the biomass, generally higher than that of PHBV, was also enhanced by the shift in feeding strategy, achieving values of 31.6 wt. % on day 268. These results could also be attributed to the increase in the COD/N ratio. However, considering the overall operation (Fig. 1c), during S-I an increase in COD/N led to higher fatty acids concentrations, whereas PHBV concentrations did not show this increase. In contrast, when the feeding strategy was modified from multiple to single pulse (S-II), the average PHBV values increased. These concentrations further increased with the raise in the COD/N ratio during S-III, demonstrating that although a higher COD/N ratio improve PHBV concentration, an appropriate feeding strategy is also crucial.

In contrast to other research works with waste fish oil [34, 39], the present study obtained better results, in terms of PHBV accumulation and biomass concentration, using a single pulse feeding, and confirmed the preference to accumulate fatty acids over PHBV when an oil is used as substrate (Fig. 1c).

### Uncoupled C and N: the challenge of pH control with oily substrates

It is known that uncoupled carbon and nitrogen feeding strategies favour the accumulation of bioproducts like PHA [30]. However, it was observed that with oily substrates, the uncoupling strategy destabilizes the pH, and acidic conditions can be generated inside the reactor [39]. In these conditions, pH control inside the biological reactor is essential. However, the addition of alkaline compounds to increase the pH can cause the saponification of the oil-based substrate, which can also lead to process failure. To manage these situations is therefore vital for the process.

In the present study, SBR1 was initially operated without pH control. During the first 170 operational days, no problems of acidification were observed (Fig. 1a). The pH was likely maintained thanks to the buffering capacity of the nutrient solution (pH fixed at 7.0 ± 0.5), which contained KH_2_PO_4_ and NaHCO_3_. Non-consumed nitrogen in the initial period might also help to maintain the pH stable. However, as nitrogen consumption improved, the pH inside the reactor at the end of the famine phase decreased to values of 3–4 (days 170–250), resulting in an acidic medium and subsequently causing microbial stress.

Some authors have reported that low pH values (< 4) can inhibit active biomass, ultimately leading to process failure [46]. Nevertheless, other studies suggest that stress conditions in short time periods, such as low pH, may promote the accumulation of PHA. Microorganisms utilize this polymer for stress resistance, as PHA monomers serve as chemical chaperones that protect proteins and other biomolecules from denaturation under stress conditions [47–49]. This factor may contribute to the observed higher PHBV accumulations on days 182 and 203 (8.9 and 19.9 wt. %, respectively). Although this pH stress may be beneficial for short-term PHA accumulation, previous studies have reported that pH values of 7.0 or even 8.5 are optimal for PHA synthesis [50, 51]. However, studies with UCO have reported that pH above or below 7, even with minimal deviations, have a negative effect on PHA production [52]. Extended periods of stress can reduce or even inhibit the growth of the culture accumulating PHA, leading to a decrease of the PHA produced [48], as evidenced by the decrease in biomass concentration in the present study due to the inability of many microorganisms to survive under such conditions.

To mitigate this issue, continuous pH control (7.0 ± 0.3) was implemented in SBR1 from day 240 onwards. After this implementation, the active biomass concentration increased again (Fig. 1a).

The pH value was set at 7.0 to provide suitable conditions for both oil hydrolysis and bacterial growth [53], as well as for the enrichment of MMCs for PHA production [50]. This value was also selected to minimize NaOH consumption during pH control and to prevent UCO saponification under alkaline conditions [54]. The use of lipid-based wastes like UCO requires a hydrolysis step to convert triglycerides into free fatty acids prior to microbial uptake. Since lipase activity is generally favored under mildly acidic to neutral conditions [53], maintaining a pH around 7.0 represented an appropriate compromise between promoting UCO hydrolysis and ensuring stable microbial activity for PHA accumulation.

### Reactor configuration: effluent withdrawal and nutrient filling

SBR2 was operated similarly to SBR1 in stage S-III, with the feeding added in a single pulse and a COD/N ratio of 46 g/g, however, SBR2 had a different reactor volume (2 L) and cycle configuration. The biomass withdrawal and refilling with a nutrient solution in SBR1 occurred during the transition from feast to famine phase, whereas in SBR2, it took place in the transition from famine to feast phase. SBR1 configuration enables the reactor to act as an enrichment and accumulation unit, as withdrawal occurs immediately after accumulation is complete. In contrast, SBR2 configuration corresponds to an enrichment reactor, and a subsequent accumulation unit will be necessary for having effluent withdrawal at the end of the famine phase, when the bioproducts inside the biomass have already been consumed. Furthermore, in SBR2, the feast/famine lengths were slightly different, 5/7 h/h between days 0 and 94; thereafter, the same values as SBR1 (6/6 h/h) were used until the end of the operation (days 95–188).

**Fig. 2:**
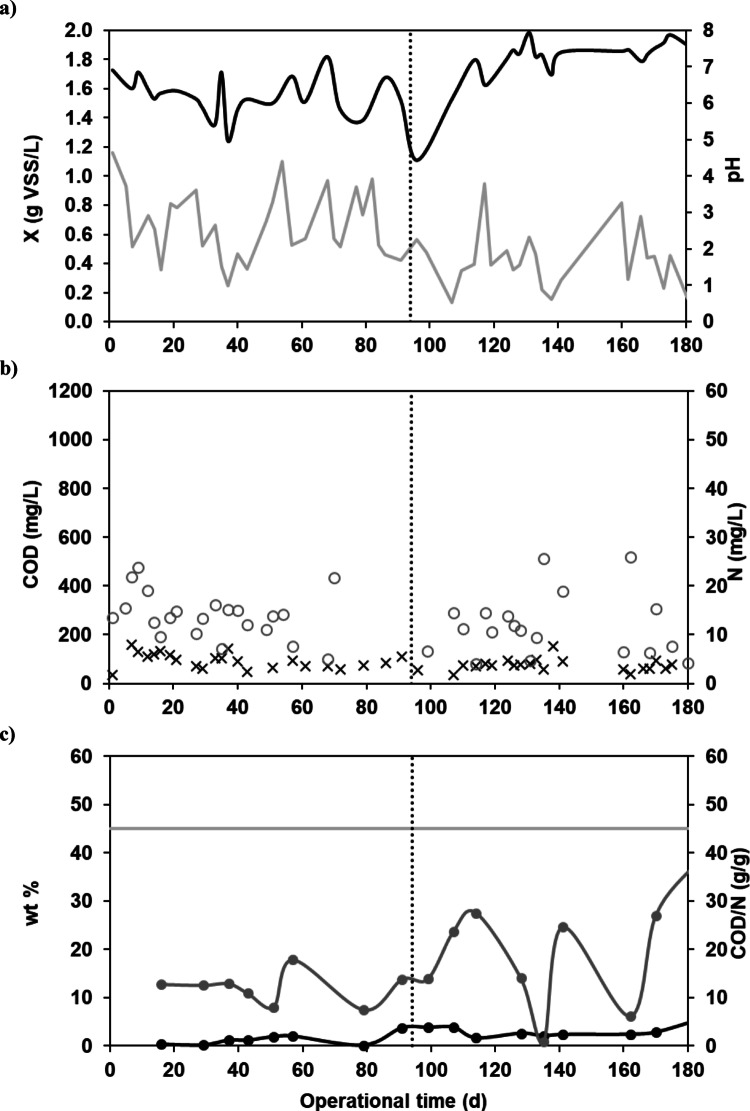
Evolution throughout the operational time of different parameters in SBR2: **a** concentration of active biomass X (), and pH value () at the end of the famine phase; **b** concentrations of sCOD at the end of the feast phase (×) and of N at the end of the famine phase (○);** c** percentages of accumulated PHA () and fatty acids () at the end of the feast phase and COD/N ratio () fed through the operation. The change in the feast/famine ratio is defined with a pointed vertical line ().

Regarding the active biomass in SBR2 (Fig. 2a), the higher values were obtained with a feast/famine length of 5/7 h/h, achieving average values of 0.6–0.8 g VSS/L at the end of the famine phase. Increasing the feast phase to 6 h (6/6 h/h), without modifying the amount of raw UCO added, decreased the active biomass concentration to values of 0.4 g VSS/L. However, this extension of the feast phase was beneficial for maintaining the pH in the reactor above neutral values.

Regarding the soluble COD values, those at the end of the feast phase in SBR2 were consistently below 100 mg sCOD/L after the first 30 days (Fig. 2b), while it took about 100 days in SBR1 to reach similar values. This could indicate that starting the SBR operation with an optimised COD/N ratio (46 g/g in SBR2), and with this enrichment configuration, can help to balance the hydrolysis and the consumption of the raw UCO in a shorter time. The nitrogen concentration at the end of the famine phase was below 10 mg N/L only on isolated days (Fig. 2b), indicating limited nitrogen consumption for biomass growth. This is likely due to the low PHA synthesis during the feast phase, which reduces the availability of stored carbon that can later be utilized together with nitrogen for biomass growth during the famine phase (Fig. 2c).

Similar to SBR1, the SBR2 resulted in a preferential accumulation of fatty acids over PHBV (Fig. 2c). However, if the maximum percentages of accumulation in SBR1 and SBR2 are compared for PHBV (19.9 and 5.2 wt. %, respectively) and fatty acids (31.6 and 37.4 wt. %, respectively), it can be concluded that the SBR1 configuration (withdrawal at the end of the feast phase) is preferable to promote PHBV accumulation. Regarding the monomeric composition the PHBV copolymer showed variations in the relative proportions of 3HB and 3HV in both units (SBR1 and SBR2), associated with operational changes, indicating that the withdrawal configuration did not play a determining role (Fig. S2).

### Reactor populations: microbiology analysis

**Fig. 3:**
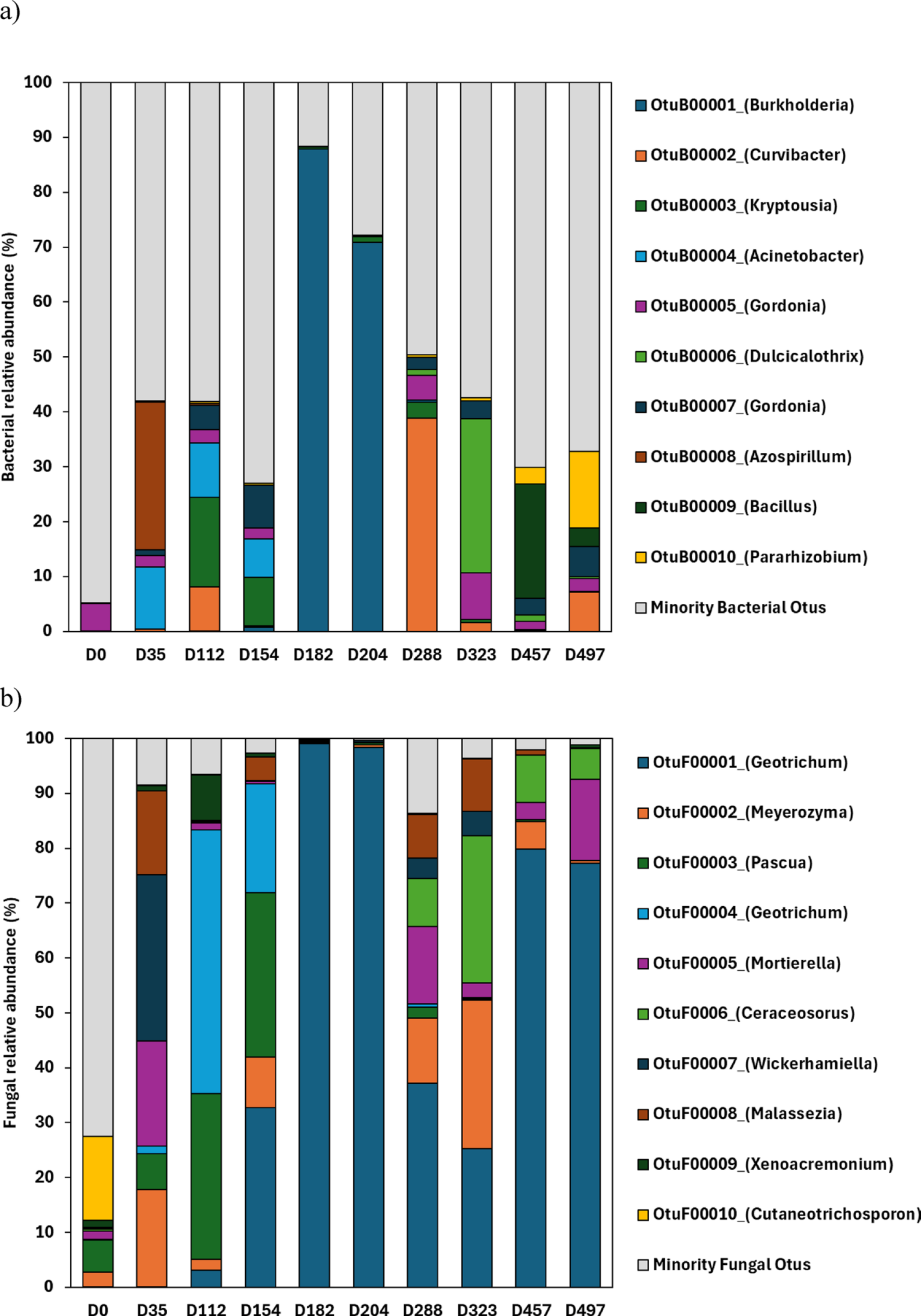
Microbial relative abundance of OTUs in SBR1 at different operational days, **a)** bacterial relative abundance and **b)** fungal relative abundance. Only dominant bacterial OTUs (relative abundance > 1 %) are shown

Significant shifts in the abundances of dominant operational taxonomic units (OTUs) for bacteria were observed throughout the operational periods, reflecting a high degree of dynamics within the MMC. Therefore, the imposed operational conditions had a strong capacity to modulate the bacterial community structure over time. The dominant OTUs observed were OtuB00001 (*Burkholderia*, 15.96 %), OtuB00002 (*Curvibacter*, 5.65 %), and OtuB00003 (*Kryptousia*, 3.03 %) (Fig. 3a).

Nevertheless, despite the instability of the bacterial population structure throughout the operation of SBR1, among the known PHA-accumulating bacteria the dominant OTUs detected at most sampling times corresponded to OtuB00008 (*Azospirillum*) [55] at day 35, OtuB00004 (*Acinetobacter*) [56] at days 112 and 154, OtuB00007 (*Gordonia*) [57] at day 154, OtuB00001 (*Burkholderia*) [58] at days 182 and 204, OtuB00009 (*Bacillus*) [59] at day 457. This broad dominance of PHA-accumulating bacteria underscores the importance of high functional redundancy within an MMC, enabling it to withstand the various stresses encountered during operation.

Furthermore, it was observed that the maximum PHBV accumulated percentage in the biomass was only achieved when the relative abundance of *Burkholderia* was coupled with *Chitinophaga*. The genus *Burkholderia* is very abundant in the environment, occupying diverse ecological niches, such as soils and waters, being one of the well-studied native PHA producers that can feed on a variety of carbon sources to produce PHA in form of 3HB and 3HV, including a copolymer of PHBV [60]. Members of this genus have the capacity to transform vegetable oils into 3HB achieving a maximum yield of 70 wt. % [58]. The genus *Chitinophaga*, comprises soil-dwelling gliding microorganisms, that produce myxospores by transforming filaments into spherical bodies, characterized by strong chitinolytic activity and contains several genes encoding glucosidase enzymes [61]. However, its ability to degrade UCO has not yet been reported.

Thus, as both genera seemed to act synergistically in the accumulation of PHBV, it could be hypothesised that *Chitinophaga* transformed the fats in this residue into free fatty acids through the action of its lipases, and *Burkholderia* finally metabolised these fats into PHBV by the PhaC enzyme, the key enzyme in the PHA biosynthesis. This close relationship is also confirmed by the NMS analysis performed that correlated the higher PHBV accumulation with higher relative abundances of *Burkholderia* and *Chitinophaga*. To confirm this relation, the Spearman rank correlation analysis was performed (Table S.4), revealing that higher relative abundances of *Burkholderia* were strongly positively related to an increase in *Chitinophaga* (ρ = 0.834), which are also correlated with higher PHBV accumulation capacities (ρ = 0.624 for *Burkholderia* and ρ = 0.803 for *Chitinophaga*). Considering that this synergetic association is first described here, the role of both bacteria should be explored to address the potential biotechnological use of these two genera as a promising alternative to enhance PHBV accumulation for the valorisation of raw UCO.

Regarding the decrease in pH in SBR1, it does not appear to hinder the development of PHA-accumulating bacteria, as a selective increase in the community was observed on days 182 and 204, resulting in the high abundance of the OTUB00001, taxonomically classified as *Burkholderia*. It should be noted that some strains of *Burkholderia* are acidophiles, capable of surviving at pH values lower than 7 [62], as is the case for those found at days 170–240. This likely promoted the growth of this bacterial genus over other bacteria less tolerant to acidic conditions. Nevertheless, the implementation of pH control from day 240 onwards did not result in the stabilisation of the bacterial populations within the MMC, as a highly dynamic community was still observed during this period.

Regarding the fungal populations (Fig. 3b), they were predominantly dominated by OtuF00001 (*Geotrichum*, 45.31 %), followed by OtuF00002 (*Meyerozyma*, 7.66 %) and OtuF00003 (*Pascua*, 7.57 %). Overall, the structures of the fungal communities were more stable than those of the bacterial communities, especially after day 182, when the community was predominantly dominated by OtuF00001 (*Geotrichum*), achieving a maximal relative abundance of 99.06 %. This dominance pattern can be attributed to the acid tolerance of this genus [63]. Although it can grow over a wide pH range (about 3 to 11), its optimal pH lies between 4 and 5.5 [64], which allows its growth once the medium becomes more acidic due to improved nitrogen consumption (days 170–240). Additionally, the production of volatile organic compounds by *Geotrichum*, particularly phenylethyl alcohol, may inhibit the growth of competing fungi through various pathways [65], that could be hypothetically exploited as an advantage to maintain its dominance over other fungi. However, this dominance was maintained even at the higher pH values observed at the end of phase S-III. Finally, it should be noted that *Geotrichum* is an oleaginous yeast [66, 67] which, could have been responsible for the content of fatty acids in the biomass of SBR1.

**Fig. 4:**
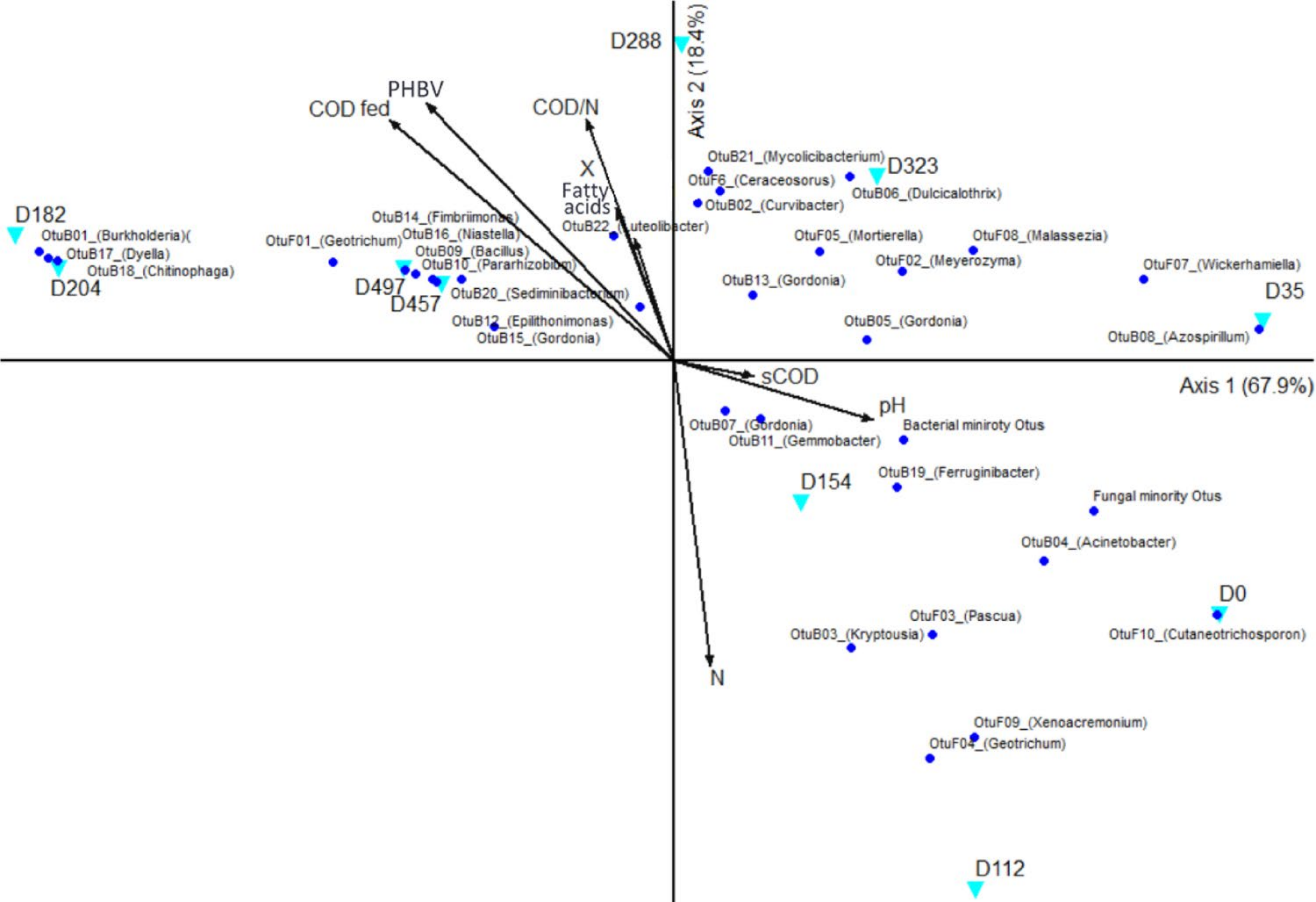
Nonmetric multidimensional scaling (NMS) ordination of dominant bacterial and fungal OTUs (RA > 1 %) found in biomass samples retrieved from SBR1, and their links with the operational variables and PHBV and fatty acids accumulation percentages. *PHBV:* % PHBV at the end of the feast phase, *Fatty acids:* % fatty acids at the end of the feast phase, *tCOD fed:* chemical oxygen demand fed as UCO, *pH:* pH in the reaction medium at the end of the famine phase, *sCOD:* soluble chemical oxygen demand at the end of the feast phase, *N:* nitrogen in the reaction medium at the end of the famine phase, *X:* active biomass at the end of the famine phase, *COD/N:* COD/N ratio fed in the cycle

The NMS vectors representing PHBV and fatty acids contents in the biomass are spatially close (Fig. 4). In this regard, *Geotrichum* (OtuF00001 and OtuF00004) has been reported to be capable of biosynthesizing enzymes with high hydrolytic capacities, the Spearman correlation analysis (Table S.4) suggests a cooperative role in PHBV accumulation, due to the strong correlation between PHBV accumulation and the relative abundances of this fungus (ρ = 0.574). Hence, the operational factors enhancing the simultaneous enrichment of *Geotrichum*, *Burkholderia*, and *Chitinophaga* needs to be thoroughly explored as an alternative for selecting a microbial community of *Bacteria* and *Fungi* that synergistically interact in the valorisation of raw UCO into PHBV.

The NMS revealed also that the main operational parameter promoting PHBV accumulation was the tCOD fed (raw UCO), suggesting that a higher OLR, and subsequently a rise in carbon availability, increased the PHBV accumulation capacity (Fig. 4). The COD/N ratio fed in each cycle was related to higher PHBV accumulations, and, on the other hand, higher N in the reaction medium at the end of the famine phase was inversely associated to larger PHBV yields, suggesting that an increase in the carbon supply and an effective reduction in the nitrogen availability is mandatory to enhance the PHBV accumulation, confirming the previously defined conditions [68]. In addition, the NMS confirmed that lower pH values in the reaction medium at the end of the famine phase for a short period of time stimulated PHBV biosynthesis, as it was experimentally observed on days 170–240, according to the protective role of PHA in bacterial fitness against various environmental stressors [69].

### Challenges of the PHA production process based on MMC and raw UCO

Despite the growing interest in replacing conventional fossil-based plastics with PHAs, their large-scale commercialization remains constrained by high production costs, which still limit their competitiveness. Several techno-economic analyses [16, 70–73] have shown that the use of MMCs can substantially reduce both capital and operational costs by (i) eliminating sterilization requirements, (ii) enabling the use of low-cost waste substrates, and (iii) integrating PHA production into waste treatment and management systems.

The use of raw UCO and MMCs for PHA production meets these requirements. However, this approach presents several challenges, including substrate complexity, conversion efficiency, and process stability. The poor solubility of UCO requires an effective hydrolysis step to release free fatty acids, which can inhibit microbial activity and compromise reactor performance. Consequently, the enrichment of PHA-storing microorganisms in MMCs fed with lipid-based substrates is typically slower and less stable than with readily biodegradable carbon sources such as VFAs. Nevertheless, the present study demonstrated that this enrichment is achievable using raw UCO without any type of pretreatment. Starting from conventional activated sludge and applying strategies like ADF and DGL, the operational conditions of the enrichment reactor (SBR1) were progressively optimized, achieving notable PHBV contents at the end of the feast phase (9 wt. % on day 182; 20 wt. % on day 203; 18.5 wt. % on day 288; and 12 wt. % on day 476), with 3HB identified as the main constituent, prevailing over 3HV in these days (Fig. S.2). The results obtained in this study, after 500 days of SBR1 operation, confirm that the enrichment of PHA-accumulating MMC with raw UCO is feasible, with key microbial populations identified as *Burkholderia* and *Chitinophaga* among bacteria, and *Geotrichum* among fungi.

In contrast, Tamang and Nogueira [27] reported that direct enrichment using raw UCO was not feasible due to the proliferation of filamentous bacteria, leading to foaming and bulking. In their study, an MMC previously enriched with nonanoic acid achieved a maximum PHA content of 38.2 wt. % from raw UCO at 40 °C, but only in a dedicated accumulation reactor. Even with pure cultures, literature reviews [10, 21, 74, 75] report a wide range of PHA contents (36–80 wt. %) when using oil-rich wastes and waste cooking oil, indicating that achieving high intracellular accumulation is not always straightforward, even with specific strains and optimized conditions.

In the present research work, the PHBV content reached in SBR1, although significant for an open culture directly fed with raw UCO, was neither stable nor sufficiently high to enable efficient extraction, which typically requires intracellular contents above 30–40 wt. % in MMCs [76]. This suggests that, although enrichment was achieved, further optimization of the accumulation process is still necessary, either by improving the operational conditions within the same reactor (for example, by extending the feast phase) or by coupling the enrichment stage with a dedicated accumulation unit.

Regarding substrate variability, although the composition of raw UCO may differ depending on the type and frequency of use, the UCO used in a potential PHA production process would be supplied by companies specialized in its collection and management. As a result, the UCO composition within a given region tends to be relatively consistent, resulting in reduced variability compared with other complex wastes that require prior fermentation to VFAs. Furthermore, the existing collection and standardization systems established for biodiesel production represent a clear advantage. Additionally, the UCO used for PHA synthesis does not require the extensive pretreatment steps typically applied for biodiesel feedstock preparation (e.g., water or solids removal). Therefore, as in the present study, pretreatment can be omitted or limited to the simple removal of coarse solids.

## Conclusions

The increase of the active biomass concentration in the enrichment reactors was successfully achieved through a progressive rise in the organic loading rate, fed as raw UCO, followed by a nitrogen limitation. A COD/N ratio around 46 g/g allowed to maintain an active biomass concentration up to 1 g VSS/L. Furthermore, when a single UCO pulse was provided, the biomass exhibits better performance in synthetizing PHBV (12 wt. %) compared to the three-pulse strategy (< 2 wt. %), which was attributed to better substrate hydrolysis and assimilation. The SBR configuration with the withdrawal and filling with nutrients at the end of the feast phase (SBR1) and 10 L of working volume, consistently reached better PHBV accumulation results (average values of 4.2 ± 1.6 wt. %, maximum of 19.9 wt. %) than the configuration with the withdrawal and filling with nutrients at the end of the famine phase and 2 L of working volume (SBR2, average values of 2.7 ± 1.1 wt. %, maximum of 5.2 wt. %). However, although a maximum accumulation of 19.9 wt. % for PHBV and 31.6 wt. % for fatty acids were reached in SBR1, process operation under these conditions was unstable, and high storage levels could not be maintained over time.

Process pH was demonstrated to be a key parameter for system operation. A transient pH decreases enhanced PHBV production due to its stress resistance function, whereas prolonged exposure to acidic conditions negatively affected microbial activity and bioproducts synthesis.

Microbial community analysis revealed a strong successional dynamic of PHA accumulating bacterial populations, primarily including *Azospirillum*, *Acinetobacter*, *Gordonia*, *Burkholderia*, and *Bacillus.* The high degree of functional redundancy within the MMC enabled resilience to operational and environmental changes, while multivariate analysis confirmed that synergistic interactions between *Burkholderia* and *Chitinophaga* were essential for the successful valorisation of UCO into PHBV.

Overall, the present study demonstrates the feasibility of producing PHBV from raw UCO using MMC, while underling the role of the operational strategy, feeding mode, and microbial interactions. Future work should focus on optimize this process stability, targeting larger active biomass concentrations and higher percentages of accumulated PHBV to support the scale-up and implementation of this method for lipid-rich waste valorisation.

## Supplementary Information

Below is the link to the electronic supplementary material.

Supplementary Material 1

Supplementary Material 2

## Data Availability

The data that support the findings of this study are not openly available but are available from the corresponding author upon reasonable request.
