# Supplementary material for: Enrichment of a mixed microbial culture for PHA production with used cooking oil as substrate
Source: Bioprocess Biosyst Eng. 2026 Jul 9;49(8):2045–59. doi: 10.1007/s00449-026-03359-x (PMC13424269; doi:10.1007/s00449-026-03359-x)
Supplement: Supplementary file 1 — Supplementary Material 1 [file 449_2026_3359_MOESM1_ESM.docx]

**Table S.1** Distribution of the SBR cycle phases over the 12-hour (in minutes).

1. SBR1 cycle distribution in stage S-I

|  | Feast phase (6 h) | | | | | | | | Famine phase (6 h) | | |
| --- | --- | --- | --- | --- | --- | --- | --- | --- | --- | --- | --- |
| Aeration |  | | | | | | | |  | | |
| UCO addition |  |  |  | |  |  | |  |  | | |
| Withdrawn |  | | | | | | | |  |  | |
| Nutrients and Nitrogen addition |  | | | | | | | |  |  |  |
| Time (min) | 3 | 87 | | 3 | 87 | | 3 | 177 | 5 | 4 | 351 |

1. SBR1 cycle distribution in stages S-II and S-III

|  | Feast phase (6 h) | | Famine phase (6 h) | | |
| --- | --- | --- | --- | --- | --- |
| Aeration |  | |  | | |
| UCO addition |  |  |  | | |
| Withdrawn |  | |  |  | |
| Nutrients and Nitrogen addition |  | |  |  |  |
| Time (min) | 9 | 351 | 5 | 4 | 351 |

1. SBR2 cycle distribution

|  | Feast phase (5 h/6 h) | | | | Famine phase (7 h/6 h) | |
| --- | --- | --- | --- | --- | --- | --- |
| Aeration |  | | | |  | |
| UCO addition |  | |  |  |  | |
| Withdrawn |  |  | | |  | |
| Nutrients addition |  |  |  | |  | |
| Nitrogen addition |  | | | |  |  |
| Time (min) | 5 | 4 | 4 | 287/347 | 1 | 419/359 |

**Table S.2** Characteristics of the raw UCO fed as the organic carbon source.

| **Parameter** | **Value** |
| --- | --- |
| Density (g/L) | 867.85 ± 33.3 |
| tCOD (g/g) | 2.63 ± 0.29 |
| Biodegradability (%) ^1^ | 58.47 ± 2.2 |
| Elemental composition (%) ^2^ |  |
| C | 76.15 ± 1.90 |
| H | 12.26 ± 0.49 |
| O | 11.13 ± 1.55 |
| N | 0.19 ± 0.09 |
| S | - |
| Fatty acid profile (%) ^3^ | 93.84 ± 2.34 |
| C-12:0 (Lauric) | < 0.01 |
| C-14:0 (Myristic) | 0.14 |
| C-15:0 (Pentadecanoic) | < 0.01 |
| C-16:0 (Palmitic) | 7.67 |
| C-16:1 (Palmitoleic) | 0.26 |
| C-17:0 (Margaric) | 0.06 |
| C-17:1 (Margaroleic) | 0.05 |
| C-18:0 (Stearic) | 3.91 |
| C-18:1 (Oleic) | 37.08 |
| C-18:2 (Linoleic) | 49.14 |
| C-20:0 (Arachidic) | 0.27 |
| C-18:3 (Linolenic) | 0.20 |
| C-20:1 (Eicosenoic) | 0.21 |
| C-22:0 (Behenico | 0.78 |
| C-22:1 (Erucic) | < 0.01 |
| C-24:0 (Lignoceric) | 0.24 |
| Others | < 0.01 |
| Trans Oleic (t-C18:1) | 0.09 |
| Trans Linoleic + Trans Linolenic (t- C18:2 + t-C18:3) | 0.25 |

^1^ Oxygen uptake rate test with the respirometer BM-T Plus 151,204 Surcis, Spain.

^2^ Elemental analyser (FlashSmart, Thermo Fisher Scientific, US) by means of a thermal conductivity detector (university internal service).

^3^ Gas chromatography in the *Instituto de la Grasa* (Sevilla, Spain). ISO 12966-2:2011 & ISO 12966-4:2015.

tCOD: total chemical oxygen demand.

| a)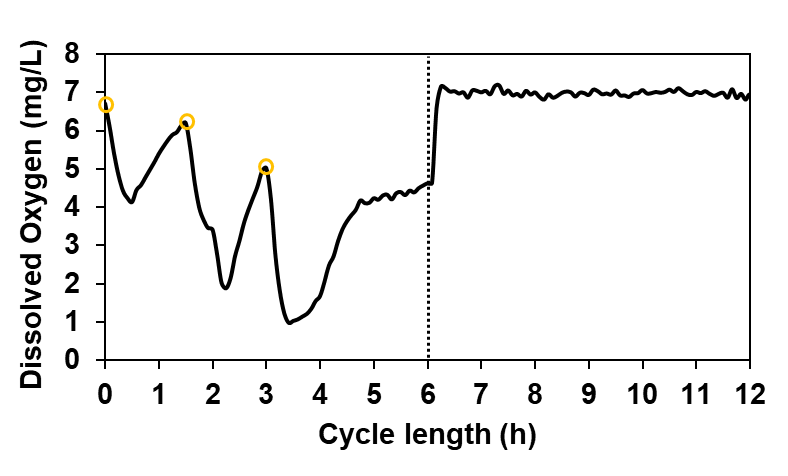 |
| --- |
| b)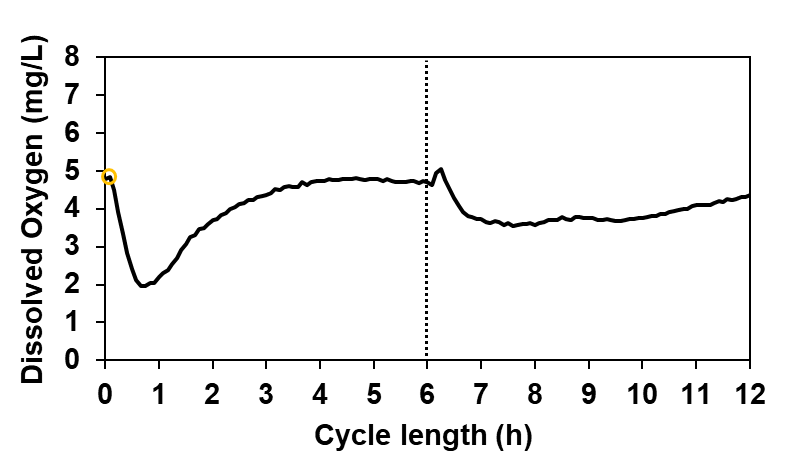 |

**Fig. S.1** Oxygen consumption through two different cycles: (a) three-pulse cycle (day 126); (b) single-pulse cycle (day 225). The moment of the UCO pulses addition (
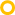
) and the separation between the feast and famine phase (⁞) are indicated in the image.


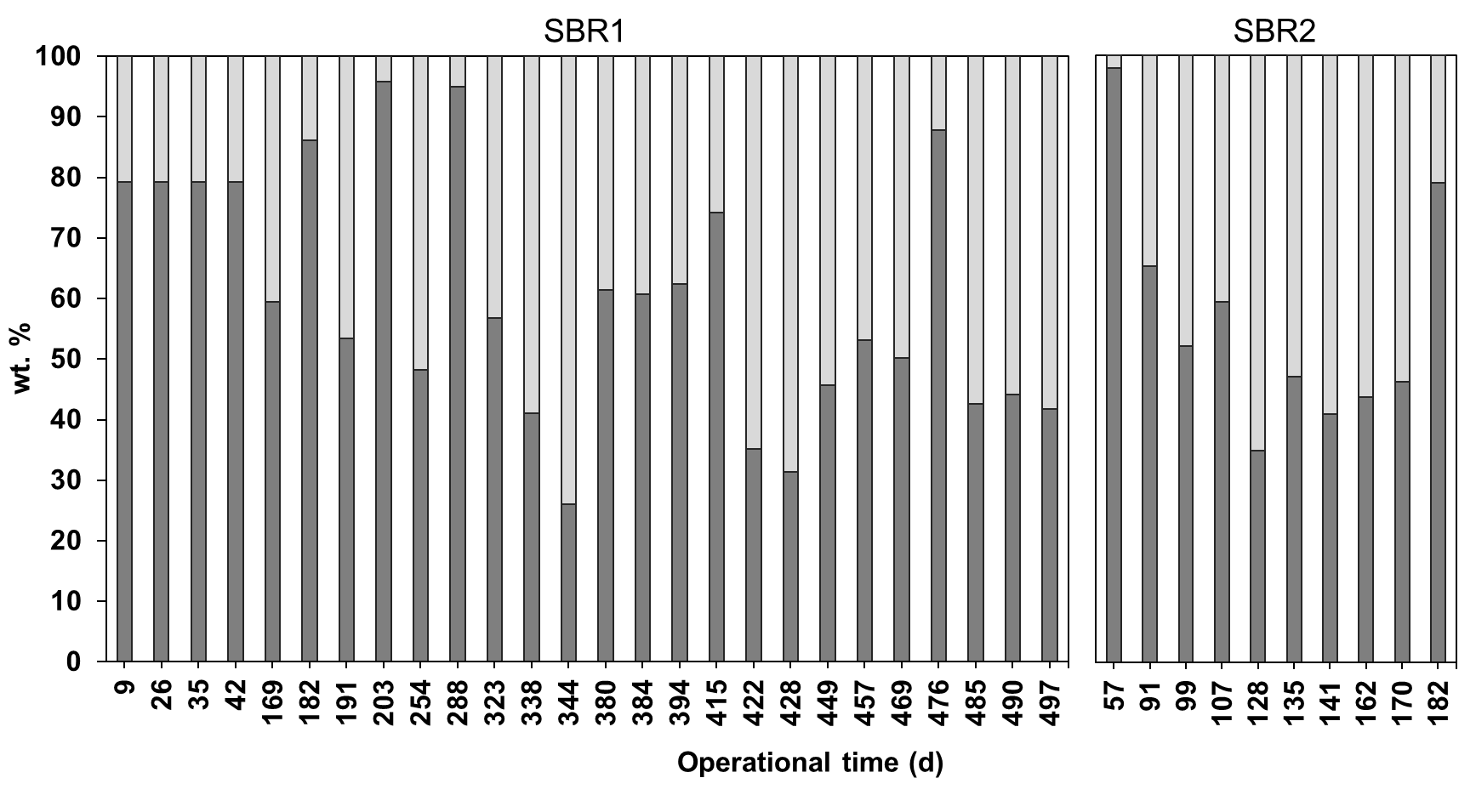
**Fig. S.2** PHBV monomer composition in wt. % with respect to the total biopolymer in SBR1 and SBR2 during the operation period: 3HB (■) and 3HV (■).

**Table S.3** Chemicals and reagents used, including the source of procurement and purity.

| **Compound** | **Purity (%)** | **Supplier** |
| --- | --- | --- |
| Ammonium Chloride (NH_4_Cl) | ≥ 99 | Sigma-aldrich, USA |
| Hydrochloric acid (HCl) | 37 | Panreac applichem, Spain |
| Sodium hydroxide (NaOH) | ≥ 99 | Sigma-aldrich, USA |
| Potassium dihydrogen phosphate (KH_2_PO_4_) | ≥ 99 | Sigma-aldrich, USA |
| Magnesium sulfate (MgSO_4_) | 96 | Sigma-aldrich, USA |
| Sodium hydrogen carbonate (NaHCO_3_) | ≥ 99 | Panreac applichem, Spain |
| Potassium chloride (KCl) | ≥ 99 | Panreac applichem, Spain |
| Allylthiourea (N-allylthiourea) | ≥ 99 | Sigma-aldrich, USA |
| 1,2-dichloroethane | ≥ 99 | Sigma-aldrich, USA |
| 1-propanol | ≥ 99 | Sigma-aldrich, USA |
| Poly(3-R-hydroxybutyrate*-co-*3-R-hydroxyvalerate) (PHBV) | 3HB: 90.8 | Sigma-aldrich, USA |
|  | 3HV: 9.2 |  |
| Palmitic acid | ≥ 99 | Sigma-aldrich, USA |
| Stearic acid | ≥ 98 | Sigma-aldrich, USA |
| Oleic acid | ≥ 99 | Sigma-aldrich, USA |
| Linoleic acid | ≥ 98 | Sigma-aldrich, USA |
| Benzoic acid | ≥ 99 | Sigma-aldrich, USA |
